# Morphine-induced hallucinations - resolution with switching to oxycodone: a case report and review of the literature

**DOI:** 10.1186/1757-1626-2-9391

**Published:** 2009-12-23

**Authors:** Mursheda Mahbub Chowdhury, Richard Board

**Affiliations:** 1 Department of Palliative Medicine, St Michael's Hospice, 25 Upper Maze Hill, St Leonard's-on-Sea, East Sussex TN38 0LB, UK; 2 St Michael's Hospice, 25 Upper Maze Hill, St Leonard's-on-Sea, East Sussex TN38 0LB, UK

## Abstract

Palliation of pain with morphine in cancer patients can be complicated by adverse effects. Tolerance to these effects such as nausea and drowsiness usually occurs within a few days allowing continuation of morphine therapy. However, some patients may develop intolerable adverse effects even after several months on morphine when the dose is increased. A case of morphine-induced hallucinations in a cancer patient who had been on a subcutaneous infusion of diamorphine for several months is discussed. A switch to oxycodone resolved his hallucinations and gave him a new lease of life. The theories behind and evidence for opioid-switching is discussed along with strategies for dealing with intolerable opioid-induced adverse effects.

## Case presentation

A 73-year-old British Caucasian gentleman, had been diagnosed with prostate cancer one year previously and was on hormone therapy. Three months after diagnosis, he was found to have a rising PSA (prostate specific antigen) and ALP (alkaline phosphatase) as well as pain in his right hip. A bone scan revealed bone metastases. Radiotherapy made no difference to the hip pain. He then tried a variety of analgesics from the family doctor including a NSAID (non-steroidal anti-inflammatory drug) and oral morphine but adequate dosing was hindered by nausea and vomiting.

He was eventually referred to a palliative care physician who commenced him on a subcutaneous infusion of diamorphine 20 mg and cyclizine 15 0 mg over 24 hours. This controlled the nausea and eased the pain although not completely.

The patient was then referred for hospice admission for respite and further symptom control. An increase in diamorphine to 30 mg over 24 hours in the syringe driver gave him good pain control. As he was doing so well, a trial of oral morphine and cyclizine was initiated. However, the nausea and vomiting recurred within a few hours and so he was recommenced on the syringe-driver, again with good effect. He was then discharged.

The patient had been reasonably symptom-controlled the first 3 weeks after discharge. Unfortunately he had then become unwell with diarrhoea and had experienced more pain. The family doctor increased the diamorphine to 50 mg in the syringe driver to control both the diarrhoea (which resolved) and the pain. However, he then became nauseated. As a result, the anti-emetic was changed to haloperidol 5 mg and then to levomepromazine (nozinan) 12.5 mg which still did not control his nausea. By then, the patient was also experiencing frightening hallucinations and he and his wife were finding it very hard to cope at home. The hospice at home team were providing bolus injections of nozinan for the nausea and hallucinations but these seemed to have little effect. He was then re-admitted to the hospice for symptom control.

Initial assessment revealed signs of opioid toxicity including meiosis and myoclonic jerks. The symptoms of nausea and hallucinations were also consistent with opioid toxicity. The patient appeared to be pain-controlled. The initial management was therefore to decrease the diamorphine in the syringe driver back down to 30 mg over 24 hours. Recent blood test results were obtained which revealed normal renal function, full blood count and calcium although liver function tests were abnormal indicating probable liver metastases and therefore disease progression. The hallucinations resolved completely and the nausea settled down with this reduction in diamorphine.

Over the next few days, the patient felt that his pain wasn't optimally controlled and so the diamorphine was cautiously increased again, this time to 40 mg over 24 hours. Initially, the vomiting recurred as a result of this change but not the hallucinations. The nozinan was increased to 50 mg to control the nausea but the patient went on to develop itch and hallucinations. He became agitated, confused with fluctuating conscious level, stopped eating and drinking and became bed-bound. He also had a disturbed sleep-wake cycle. These are the features of delirium as required by the ICD-10 (International Classification of Diseases, 10^th ^revision, WHO 1993). The differential diagnoses at this point included delirium secondary to opioid toxicity and delirium as a feature of pre-terminal agitation. In favour of the former were his known sensitivity to morphine as evidenced by the previous improvement in his symptoms with dose reduction. In favour of the latter were his weight loss (cancer cachexia) noted since his previous admission and the deterioration in his blood tests (liver function) indicating liver metastases. There was no evidence of sepsis, hypoxia, drug interaction or other co-morbidities that could account for the delirium.

The diamorphine 40 mg was then switched to oxycodone 30 mg and within 24 hours the hallucinations, agitation and nausea resolved. Over the next few days, he continued to improve. The nozinan in the syringe driver was reduced to a less sedative dose and he went home 10 days after the opioid switch. He felt he had his quality of life back - "I feel alive again" and enjoyed the next 3 months, which included Christmas at home with his family.

## Discussion

This case illustrates the potential for morphine to cause intolerable side-effects which can be resolved by a switch to an alternative opioid. As delirium in cancer patients can be secondary to a variety of causes (Table 1), it is important to consider opioid-induced delirium in the differential diagnosis. A single patient may have one or more co-morbidities that mimic opioid-induced adverse effects. His liver function was abnormal and symptoms of drowsiness, cognitive impairment, nausea, vomiting and myoclonus are all seen in liver failure. However, this is uncommon in metastatic disease alone unless there are very extensive hepatic metastases or biliary obstruction.

**Table 1:** Co-morbidities that cause cognitive impairment and hallucinations

Metabolic	Hypoxia, hypercalcaemia, hyponatraemia, renal failure, liver failure, dehydration
**Drug-induced**	Opioids, benzodiazepines, tricyclics, corticosteroids, chemotherapy, drug withdrawal

**Recreational drugs**	Including alcohol and alcohol withdrawal

**Infection**	Particularly Urinary Tract Infection in the elderly

**CNS**	Cerebral metastases, Leptomeningeal metastases, cerebrovascular incident, head injury

**Terminal phase**	Pre-terminal agitation is a recognised phenomenon

Cherny et al (2001) reported on the recommendations of the Expert Working Group of the European Association of Palliative Care on the management of the Adverse Effects of oral morphine [1]. As the principles are the same, these recommendations could be applied to subcutaneous morphine or diamorphine or indeed any other opioid causing adverse effects. The box below summarises their recommendations.

• Careful evaluation to distinguish between morphine-induced adverse effects from co-morbidities that mimic these effects.

• Consider reduction in dose of morphine +/- adding in co-analgesics or treatments targeting the specific pain syndrome (such as radiotherapy).

• Symptomatic management of the adverse effect using neuroleptics such as haloperidol or benzodiazepines such as midazolam.

• Switching the route of opioid administration - though the authors point out there is no strong evidence for this.

• Opioid switching such as from morphine to oxycodone, fentanyl or methadone, thus reducing the risks of polypharmacy.

Applying the above strategy to his case, other causes for delirium were initially sought, and a dose reduction of morphine was made. Co-analgesia with diclofenac (a NSAID) had already been tried with little effect on overall pain control as had radiotherapy which had also made no difference. When adverse effects recurred as the morphine dose was increased, symptomatic management of these effects with nozinan and midazolam was attempted. When this strategy failed, a switch in opioid to oxycodone produced dramatic improvement. Considering an opioid switch earlier in the patient management would have, in hindsight, been less traumatic for both the patient and his family. It may be a better strategy to perhaps switch opioids if adverse effects are not subsiding within 48 hours and are causing distress to the patient. A study by Bruera et al (1995) showed a reduction in the incidence of delirium in cancer patients on opioids and a reduction in the use of drugs to manage this adverse effect when a policy of cognitive monitoring, hydration and opioid switching was introduced [2].

Most experts, including Hanks et al (2005) in the Oxford Textbook of Palliative Medicine, agree that tolerance to the common adverse effects of opioids such as nausea, sedation and mild cognitive impairment occurs within days [3]. It is therefore established practice to forewarn and forearm the patient prior to initiating or increasing the dose of opioids that these side effects are to be expected and are usually transient. Anti-emetics are usually co-prescribed. However, CNS-excitatory adverse effects such as delirium, hallucinations and myoclonus may have a dose-response relationship and a chronicity-response relationship to morphine. According to expert opinion, prolonged exposure to morphine decreases the tendency to develop sedation and respiratory depression but increases the tendency to delirium and myoclonus. In a small proportion of patients, the common side effect of nausea continues to be a problem. In this case, nausea secondary to morphine was a continuing problem, only really controlled when morphine (in the form of diamorphine) and an anti-emetic was administered via the subcutaneous route. When the dose of diamorphine was increased, the nausea recurred and could not be controlled with anti-emetics. The patient had been on diamorphine for some months when the adverse effects of delirium and myoclonus occurred and there certainly was a dose-response relationship to these effects.

### The evidence for opioid switching

A report of five cases by Galer et al (1992), demonstrated the inter-individual variability in response to different opioids [4]. In all five cases, a switch to an alternative opioid resulted in better pain control and reduction of intolerable side-effects. They suggested that a person's response to opioids is dependent on a number of variables:

(1) Pain characteristics (eg. neuropathic, bone)

(2) Drug characteristics (eg. pharmacokinetic and pharmacodynamic properties, interaction with other drugs)

(3) Individual characteristics (eg. age, genetic variation in receptor subtypes, co-morbidities)

### Pain characteristics

It is universally acknowledged that the analgesia needs to target the pain syndrome. For instance neuropathic pain is not always opioid-responsive and co-analgesics may obviate the need for dose escalation of opioid.

### Drug characteristics

The metabolism of morphine is via the UGT (uridine-diphosphoglyceryl transferase) system in the liver where the active metabolites M6G (morphine 6-glucuronide) and M3G (morphine 3-glucuronide) are produced. M6G has been shown to accumulate in renal failure and has been implicated in toxicity. M3G has been implicated in causing opioid-induced hyperalgesia and convulsions and is known to accumulate in cerebrospinal fluid in rodent studies. Extrapolating from this, it may be that M3G may be responsible for other neuroexcitatory effects such as hallucinations and myoclonus. de Stoutz et al (1995) based their study of 191 cancer patients treated with morphine, on the hypothesis that morphine metabolites were involved in the development of dose-limiting toxicity [5]. 42% of these patients required opioid switching.

Oxycodone like codeine, on the other hand, is metabolized in the liver by the enzyme cytochrome P450 2D6 (CYP2D6) to oxymorphone and by N-demethylation to noroxycodone, although it is oxycodone that is mostly responsible for both side effects and analgesia. It is postulated that the differences in metabolism of opioids may, in part, account for the differences in incidence of adverse effects and development of tolerance to these effects. A more detailed account of the pharmacological basis for opioid switching is to be found in the paper by Ross et al [6].

Different opioids bind differently to the opioid receptors. Morphine, oxycodone, methadone and fentanyl, are all μ opioid receptor agonists. Oxycodone also acts on the κ opioid receptor. These differences may also play a part in response to opioids.

### Individual characteristics

Riley et al (2004) conducted a retrospective study which aimed to identify individual characteristics including haematological and biochemical parameters that could predict morphine intolerance and the need for opioid switching [7]. They identified age > 78 years, high white cell count (>7 × 10^9^/l), high platelet count (>210 × 10^9^/l) and poor renal or liver function as important predictors. The patient only fell into the last category with an ALP of 844 iu/l and an ALT of 451 iu/l.

Inter-individual genetic variation in the genes coding for CYP2D6 and other enzymes involved in drug metabolism may account for inter-individual differences in response to opioids. It is well-established that polymorphisms in the gene for CYP2D6 can lead to poor metabolism of codeine to its active metabolite morphine. 5-10% of Caucasians lack these enzymes. Maddocks et al (1996) conducted a study evaluating the effect on morphine-induced delirium by switching to subcutaneous infusion of oxycodone [8]. He identified whether these patients were poor metabolizers or not through the dextromethorphan test. Unsurprisingly, the poor metabolizer identified required higher doses of oxycodone. Overall, his study of 13 patients found that a switch to oxycodone significantly improved mental state.

According to Ross et al (2006), genetic variation leading to variation in opioid receptor morphology and subsequently drug binding, could account for inter-individual differences [6]. Polymorphisms in the gene for the μ-opioid receptor can, in theory, alter the affinities of different opioids to the receptor. However, this needs further study as there is conflicting evidence thus far. Studies on human post-mortem brain samples reveal the wide inter-individual variation in the densities of the μ-opioid receptors which again could contribute to inter-individual differences in response to opioids.

Ross et al (2006) also discuss the role of other genes such as the MDR-1 gene which codes for P-glycoprotein, a membrane-bound transporter protein that regulates drug absorption and excretion [6]. In P-glycoprotein knockout mice, high CNS concentrations of morphine, fentanyl and methadone were measured. Likewise, similar experiments on mice revealed the critical role of other proteins such as β-arrestin-2, an intracellular protein that regulates receptor desensitization and internalization. The analgesic effect of morphine was found to be prolonged in knockout mice. Polymorphisms in these genes in human subjects would therefore be expected to influence individual responses to drugs.

### Oxycodone as the second-line opioid

Cairns (2001) reviewed the studies evaluating the use of oxycodone compared with morphine and concluded that the analgesic effects were comparable but that a switch to oxycodone should be considered in patients experiencing intolerable side-effects such as hallucinations [9]. However, there are no randomised controlled trials to support the use of oxycodone as opposed to morphine and the studies thus far were inadequately powered with small numbers of patients.

Since then, Riley et al (2006) conducted a study of 186 cancer patients treated with oral morphine for cancer pain, and found that 25% experienced either intolerable side-effects or inadequate pain relief and needed a switch in opioid [10]. Oxycodone was used as the second-line opioid and 79% of non-responders to morphine had their pain and adverse effects successfully controlled by oxycodone. This evidence further strengthens the case for oxycodone as the second-line opioid of choice. In addition, its availability in both IR and MR preparations and as a subcutaneous injection makes its use more practicable than fentanyl or methadone. These latter opioids also play important roles in pain-control, particularly in patients with renal failure in the case of fentanyl and neuropathic pain in the case of methadone, and also would be ideal candidates as third-line and fourth-line opioids in the management of inadequate pain relief and adverse effects from morphine and oxycodone. A small study by McNamara (2002) found there was an improvement in cognitive function when patients were switched from morphine to transdermal fentanyl [11]. Indelicato and Portenoy (2003) describe the case of a cancer patient with intractable, worsening pain, no longer controlled by morphine who rotated to several different opioids including fentanyl and oxycodone, but eventually found pain relief with a switch to methadone [12].

## Conclusion

In the treatment of cancer patients experiencing hallucinations, cognitive impairment and/or nausea, it is important to consider morphine-induced adverse effects as a cause, after excluding co-morbidities that may mimic these symptoms. A patient can develop CNS-excitatory side effects even after many months of morphine use, particularly when the dose is increased. A switch to oxycodone must be considered if symptoms do not subside or are not easily controlled, sooner rather than later, particularly if the patient is very distressed by the adverse effects.

## Consent

Written informed consent was obtained from the patient for publication of this case report. A copy of the written consent is available for review by the Editor-in-Chief of this journal.

## Competing interests

The authors declare that they have no competing interests.

## Authors' contributions

Both MMC and RB were major contributors in the care and management of the patient. MMC was the main contributor in the writing of the manuscript. All authors read and approved the final manuscript.
